# Understanding the influence of AI labels on consumer psychology: the moderating role of product type

**DOI:** 10.3389/fpsyg.2026.1878095

**Published:** 2026-07-02

**Authors:** Xiaolian Cheng, Inwoo Nam

**Affiliations:** Department of Business Administration, Chung-Ang University, Seoul, Republic of Korea

**Keywords:** algorithm aversion, artificial intelligence, eeriness, framing effect, labeling, perceived risk, product type, self-expression

## Abstract

Driven by rapid progress in generative artificial intelligence, the use of AI-generated models in online product displays has become increasingly widespread. Although AI-generated content appears highly realistic, it is commonly associated with adverse consumer responses. This study examines how labeling a model in a product image as AI-generated (vs. human) influences consumers' self-expression. The findings show that labeling a model as AI-generated functions as an emphasis-framing cue that elevates consumers' sense of eeriness, which subsequently increases both perceived psychological risk and perceived performance risk, thereby reducing consumers' self-expressive tendencies. This process is moderated by product type, such that the negative effect is amplified for symbolic products, whereas it disappears for functional products. Building on theories of framing effect, algorithm aversion, emotion, and risk perception, this study elucidates how AI-generated labels shape consumers' self-expression and identifies the boundary conditions of these effects across different product types.

## 1 Introduction

AI-generated technologies are increasingly being applied in online retail and digital marketing contexts, particularly in product presentation and visual display (Zhang and Hur, 2025). A growing number of brands are adopting AI-generated models to replace traditional human models in order to reduce costs and improve display efficiency (Kiper and Liang, 2026). At the same time, several countries or regions have begun to require the labeling or disclosure of AI-generated content in specific contexts, such as the European Union’s AI Act, California’s AI Transparency Act and China’s Measures for the Labeling of AI-Generated Synthetic Content (Lund et al., 2025). However, despite the high level of visual realism achieved by AI-generated images, consumer responses to such content are not entirely positive (Baek et al., 2026).

This study posits that consumers’ judgments are not solely based on the visual stimulus itself but are also significantly influenced by source-related information. Prior research indicates that when individuals encounter entities that are humanlike yet not genuinely human, they tend to experience a negative emotion known as eeriness (Ho and MacDorman, 2010), which arises from the blurred boundary between human and non-human categories (Kätsyri et al., 2015). In AI-related contexts, even in the absence of noticeable visual imperfections, the “AI-generated” label may lead consumers to perceive the image as an unnatural artifact, thereby heightening perceptions of unnaturalness and eliciting eeriness (Ho and MacDorman, 2010). According to the affect-as-information theory (Clore et al., 2012), individuals use their current emotional states as important cues in evaluating external objects. Thus, when consumers experience eeriness, this discomfort is often interpreted as a signal that something may be wrong with the product itself, leading to elevated levels of perceived risk (Sjöberg, 2007). On the one hand, consumers may construe this uneasy feeling as an indication that the product is incompatible with their preferences, thereby increasing perceived psychological risk (Kamalul Ariffin et al., 2018). On the other hand, the sense of unnaturalness may also be interpreted as a signal of potential instability in product quality or performance, thereby heightening perceived performance risk (Phamthi et al., 2024).

In the context of fashion and appearance-related products, consumption is not merely a functional choice but also a critical means through which individuals construct identity and engage in self-presentation (Çili and Khadaroo, 2026). When consumers experience eeriness induced by AI labeling, they are likely to perceive higher levels of both psychological and performance risks, which in turn reduces the product’s potential to serve as a vehicle for self-expression (Li et al., 2026). If AI-generated content undermines consumers’ ability to view a product as an extension of the self, its impact may extend far beyond simple purchase decisions and may give rise to broader negative psychological and behavioral consequences. However, existing research on AI in marketing has largely focused on outcome variables such as purchase intention or trust, with relatively limited attention devoted to how AI technologies influence consumers’ self-expression (Park and Ahn, 2024; Wu et al., 2025; Zhou and Lu, 2025). Moreover, consumers’ evaluation criteria and psychological responses systematically differ across product types (symbolic vs. functional) (Che et al., 2025; Taufik et al., 2022; Wang et al., 2024), which may further influence the effect of AI model labeling.

Although prior research has examined the impact of AI technologies on consumer decision-making, there remains a lack of systematic investigation into how AI influences self-expression through framing effect, emotional responses and risk perceptions, as well as how these effects vary across product types. Accordingly, two important yet underexplored questions arise. When the same product display is labeled as “AI-generated” rather than “displayed by a human model,” how does this source cue influence consumers’ self-expression through eeriness and multidimensional risk perceptions? Furthermore, under what product conditions is this process more pronounced?

## 2 Theoretical background and hypothesis development

### 2.1 The role of self-expression in fashion consumption

Self-expression refers to the process through which individuals construct, sustain, and communicate their self-concept through consumption choices, a view that is largely grounded in the extended self-perspective (Belk, 1988; Sirgy, 1982, 1986). In the present study, self-expression captures consumers’ willingness to use the product to convey their self-image and desired identity (van der Westhuizen and Kuhn, 2024). This construct differs from attitude, which reflects consumers’ evaluative response to the product (Koc et al., 2025); trust, which concerns judgments of reliability (Chandrruangphen et al., 2022); and purchase intention, which reflects transactional willingness (Spears and Singh, 2004). Rather than capturing general product evaluation or purchase likelihood, self-expression focuses on the perceived fit between the product and consumers’ identity-expression goals (Kaur and Anand, 2021).

Self-expression is particularly salient in the context of fashion consumption (Upadhyay and Kamble, 2025). Fashion products are directly involved in bodily presentation and are often visible to others in everyday interactions and public settings, which makes them inherently high in social visibility (Çili and Khadaroo, 2026). Consumers’ choices of fashion products therefore reflect not only preferences for style but also the management of personal taste and social identity (Helmy et al., 2026). For instance, consumers may express themselves by wearing clothing with conspicuous brand logos (Chen et al., 2026), attaching personalized charms such as Labubu-style figures to their bags (Yang and Li, 2025), or carrying trend-driven daily objects such as large-capacity Stanley cups (Pan, 2024). These practices collectively suggest that consumers use fashion-related meanings to interpret the relationship between the self and society (Morgan and Townsend, 2022) and actively rely on brands and products to communicate their self-concept (Chernev et al., 2011). Furthermore, when consumers perceive a product as capable of expressing the self, they may derive conspicuous value from it (Allen et al., 2008), develop stronger emotional attachment to the product (Siddique and Rajput, 2022), show greater brand preference, and become more likely to form a long-term loyal relationship with the brand (Chen and Liao, 2023; Kim et al., 2001).

Conversely, a lower tendency toward self-expression may give rise to a series of consumer avoidance responses (Li et al., 2026) and, in more severe cases, brand hate (Atwal et al., 2022). Product presentation subjects, such as endorsers or models, serve as important reference points through which consumers assess a product’s fit with their self-identity (Nichols and Schumann, 2012). Consumers typically infer the style associated with the product from the presentation subject and then project themselves into the depicted usage context by imagining how they would use or wear the product (Liang et al., 2022). When the meaning conveyed by the presentation subject is inconsistent with consumers’ self-image, consumers may actively avoid products that fail to represent who they are (White and Argo, 2009). For example, when a brand endorser becomes involved in negative publicity, the unfavorable meanings associated with the endorser may transfer to the brand, thereby reducing consumers’ willingness to use the brand for self-expression (Breberina et al., 2022). In a more severe case, Dolce and Gabbana’s cross-cultural marketing campaign adopted culturally negative stereotyped portrayals through its product presentation, which triggered extensive negative reactions on social media and contributed to the formation of brand hate (Atwal et al., 2022).

Furthermore, this study argues that an AI model label functions as an emphasis-framing cue (Chong and Druckman, 2007) that increases the weight consumers assign to the source of product presentation when judging whether the product fits the self. Framing theory suggests that different presentations of the same object or event can lead individuals to form different cognitions and responses (Entman, 1993; Goffman, 1974; Tversky and Kahneman, 1981). Emphasis framing works by highlighting a specific evaluative dimension, directing greater attention to that dimension, and increasing its influence on subsequent judgment (Chong and Druckman, 2007). In the present context, the AI model label serves as an emphasis frame that brings the otherwise less prominent model-source cue, namely whether the model is a real human, to the foreground. As a result, consumers may no longer focus only on the product’s overall aesthetics, quality, and price when evaluating it (Thøgersen et al., 2012). When consumers’ attention is directed to the fact that the product is displayed by an AI model, the model may be interpreted as an algorithmically generated rather than a real human bodily representation (Audrezet et al., 2025). Because fashion products rely heavily on bodily imagination and human reference points (Kaur and Anand, 2021), the AI model label may weaken consumers’ ability to imagine a realistic wearing situation and to project themselves into the product-use context. In addition, the explicit AI cue may remind consumers of prior negative experiences or concerns associated with AI, thereby triggering psychological defensiveness (Yang et al., 2023). Consequently, even when consumers find the product somewhat attractive, they may become less willing to use it as a means of expressing their self-image and personal style. By contrast, a human model is more likely to be perceived as a socially grounded and authentic presentation subject (Poirier et al., 2024), which can more readily support consumers’ self-projection and self-expression.

*H1:* Compared with when the model is labeled as a human, labeling the product display as “displayed by an AI-generated model” will lead to a lower level of self-expression.

### 2.2 The mediating role of eeriness

The uncanny valley effect refers to the phenomenon whereby non-human entities that closely resemble real humans, yet remain imperfectly human, evoke a strong sense of eeriness (Mori, 1970). Mori et al. (2012) further suggest that the aversive reactions elicited by such humanlike entities may stem from a subconscious fear of death, as these entities often lack vital signs, exhibit rigid postures, and resemble human corpses. Beyond fear, cognitive conflict arising from the coexistence of humanlike and non-human characteristics represents another underlying mechanism. Specifically, individuals may experience difficulty in categorizing such entities as either human or non-human, leading to processing fluency disruptions and psychological discomfort (Kätsyri et al., 2015). Moreover, from the perspectives of medical cognition and evolutionary psychology, anomalous features of non-human entities may be implicitly interpreted as signals of pathogen threat or disease risk, thereby triggering instinctive avoidance responses (Diekhof et al., 2025).

Eeriness represents the core negative emotional response in uncanny valley theory, reflecting individuals’ reactions when encountering humanlike yet non-human entities (Ho and MacDorman, 2017). Prior research has shown that when virtual characters exhibit subtle unnatural features in their appearance or facial expressions, consumers tend to experience greater discomfort and evaluate them more negatively (Gutuleac et al., 2024). This effect has been attributed to factors such as heightened sensitivity to unnatural details, implicit expectations of human appearance templates, and the psychological detection of technological artifacts (Miller et al., 2023). However, in these studies, AI-generated humanlike entities could still be distinguished, to some extent, through visual cues, and eeriness was therefore largely attributed to minor distortions in the image itself (Gutuleac et al., 2024; Miller et al., 2023). Importantly, with the advancement of generative AI technologies, consumers are now increasingly unable to distinguish AI-generated images from real ones based solely on visual information (Högemann et al., 2025). This suggests that eeriness does not arise entirely from the objective visual characteristics of the stimulus. Under such conditions, a critical question emerges: when visual differences are no longer salient, why does eeriness persist? This study argues that this phenomenon should be explained from the perspective of consumers’ subjective cognition of AI. Specifically, the “AI-generated” label constitutes a salient source cue (Lim and Schmälzle, 2024). When consumers become aware that a piece of content is AI-generated, they may recall prior negative experiences and stereotypes associated with AI, such as perceptions of unreliability, lack of emotion, or mechanical qualities (Yang et al., 2023).

We can use algorithm aversion to explain why people hold negative views toward AI (Mariadassou et al., 2024). Algorithm aversion refers to individuals’ tendency to be less willing to rely on or trust algorithmic decisions compared with those made by humans, even when algorithms objectively perform better (Dietvorst et al., 2015). Prior research has shown that once individuals become aware that a decision or piece of content is generated by an algorithm, their evaluations of the outcome systematically decline, and they exhibit greater sensitivity to potential errors (Filiz et al., 2023). When a product display is labeled as “displayed by an AI-generated model,” this cue may trigger negative attributions toward the algorithmic agent (Mariadassou et al., 2024), such as perceptions of lacking intentionality, emotional capacity, and social attributes (Dunham et al., 2025; Kim and Im, 2026). This attribution process can amplify consumers’ sensitivity to subtle unnatural features in the visual stimulus, even when such discrepancies are not objectively salient. In contrast, when the same image is labeled as “displayed by a human model,” consumers are more likely to interpret it as a natural representation of a real individual, thereby reducing negative emotional responses. Accordingly, we propose the following hypothesis:

*H2:* Compared with when the model is labeled as a human, labeling the product display as “displayed by an AI-generated model” increases eeriness, which in turn reduces consumers’ self-expression.

### 2.3 Perceived psychological and performance risk

In consumer behavior research, perceived risk is widely regarded as a key antecedent of consumer decision-making, reflecting individuals’ subjective expectations of potential negative outcomes under conditions of uncertainty (Featherman and Pavlou, 2003). When making purchase decisions, consumers not only consider the potential benefits of a product but also evaluate the possible losses and uncertainties associated with it (Zhang and Yu, 2020). Perceived risk is inherently multidimensional, comprising several distinct types, commonly including performance risk, psychological risk, and social risk (Bhukya and Singh, 2015; Ha, 2002). Among these, perceived psychological risk and perceived product performance risk are particularly salient in online shopping contexts (Kamalul Ariffin et al., 2018; Phamthi et al., 2024).

Perceived psychological risk refers to consumers’ subjective expectation that purchasing a product may negatively affect their self-image consistency or psychological comfort (Kamalul Ariffin et al., 2018). The model source information itself may directly increase consumers’ perceived psychological risk. Research on algorithm aversion has found that AI generation is more likely to be interpreted as a signal of unreliability (Altay and Gilardi, 2024). Even when there is no obvious problem with the product itself, the “AI-generated model” label may lead consumers to worry that the product cannot meet their needs and that purchasing the product may bring psychological burdens such as anxiety or regret, thereby increasing their perceived psychological risk. At the same time, the AI model label may further influence this psychological risk through eeriness. Prior research suggests that emotions are not merely by-products of risk judgments but constitute a critical input into risk perception (Abikari, 2024). According to the affect-as-information theory (Clore et al., 2012), when individuals face uncertain objects, they rely on their emotional states to infer whether the object is safe or appropriate, thereby transforming subjective feelings into expectations about potential outcomes. When individuals experience negative emotions such as discomfort, these feelings are interpreted as warning signals, indicating that the current situation may involve potential problems and thus increasing sensitivity to risk (Sjöberg, 2007). Therefore, when consumers experience eeriness when facing a product display labeled as using an AI-generated model, this eeriness may be interpreted as a negative warning signal, indicating that purchasing the product may involve potential problems. In other words, before actually purchasing the product, consumers have already perceived the psychological risk of worrying that the product may not meet their own needs. As this perceived psychological risk increases, consumers will suppress their tendency to engage in self-expression through the product.

*H3*: Compared with when the model is labeled as a human, labeling the product display as “displayed by an AI-generated model” increases consumers’ perceived psychological risk, which in turn reduces their self-expression.

*H4*: Eeriness and perceived psychological risk serve as sequential mediators in the relationship between model presentation source (AI vs. human) and consumers’ self-expression.

Perceived product performance risk refers to consumers’ uncertainty regarding whether a product can fulfill its expected functions or perform as intended (Phamthi et al., 2024). Unlike psychological risk, which centers on internal affective responses, performance risk emphasizes the product’s objective quality and functional reliability (Phamthi et al., 2024). Compared with a product image labeled as displayed by a human model, a product image labeled as displayed by an AI-generated model is more likely to make consumers doubt whether the display truly and accurately reflects the product’s actual usage effect. Under such conditions, even in the absence of explicit negative evidence, consumers may adopt conservative or unfavorable inferences about product quality (Ommen et al., 2010). Moreover, emotions not only shape subjective experiences but also influence information processing, thereby affecting consumers’ evaluations of product performance (Chaudhuri, 1998; Naqvi et al., 2006). Prior research indicates a significant negative relationship between perceived performance risk and product trust (Hong, 2015). When consumers believe that a product may not perform reliably, they are less likely to form favorable quality judgments and are more inclined to avoid such options to minimize potential losses (Phamthi et al., 2024). Therefore, when the AI model label, as source information that appears humanlike but is not actually human, elicits consumers’ eeriness, this feeling does not remain merely at the emotional level. Instead, consumers may use it as a diagnostic cue for judging product reliability. Specifically, when consumers experience eeriness due to the AI model label, they may further question the authenticity of the product display image, and consequently doubt whether the product itself can perform as shown in the display. As a result, eeriness may lead consumers to make more negative inferences about the product’s actual performance, increase their perceived product performance risk, and ultimately reduce their likelihood of using the product for self-expression.

*H5*: Compared with when the model is labeled as a human, labeling the product display as “displayed by an AI-generated model” increases consumers’ perceived product performance risk, which in turn reduces their self-expression.

*H6*: Eeriness and perceived product performance risk jointly exert a serial mediating effect in the relationship between model presentation source (AI vs. human) and consumers’ self-expression.

### 2.4 The moderating role of product type

Symbolic products are those that primarily satisfy consumer needs through their symbolic meanings, social status, or capacity for self-expression, such as luxury goods and fashion brands (Ang and Lim, 2006). In contrast, functional products are designed to fulfill utilitarian needs, emphasizing product performance, quality, and efficiency, such as home appliances and everyday consumer goods (Bhat and Reddy, 1998). The key distinction lies in that symbolic products emphasize emotional and social value, whereas functional products focus on practicality and functional utility (Candi et al., 2017).

Prior research suggests that different promotional and evaluative mechanisms operate across product types. In the field of digital branding and marketing, studies on brand–influencer congruence show that functional congruence tends to influence brand attitudes through perceived certainty, whereas image congruence operates through affective responses such as pleasure (Che et al., 2025). Overall, symbolic products rely more heavily on subjective factors such as social recognition and emotional resonance in driving consumer evaluations (Wang et al., 2024). In contrast, consumers evaluating functional products tend to place greater emphasis on objective information, including product specifications, quality, and price, and therefore respond to marketing cues in a more deliberate and rational manner (Baek and Kim, 2022; Dhar and Wertenbroch, 2000). Price discounts have been found to be less effective for symbolic brands but more effective for functional brands (Li et al., 2015).

In the context of symbolic products, consumers rely more heavily on visual presentation, human embodiment, and identity-related cues to infer the product’s expressive meaning (Ang and Lim, 2006). A symbolic product is not evaluated only as a physical object, but also as a vehicle for communicating style, taste, and desired identity (Wang et al., 2024). Therefore, when such a product is displayed with an AI-generated model label, the label functions as an emphasis-framing cue that makes the model’s nonhuman source more salient and more diagnostic for product evaluation (Chong and Druckman, 2007). Because the model serves as an important carrier of the product’s symbolic meaning (Nichols and Schumann, 2012), the artificiality and nonhumanness associated with the AI-generated model (Audrezet et al., 2025) are more likely to spill over to the displayed product. As a result, consumers may experience stronger eeriness, perceive greater psychological risk, and infer higher performance-related risk in terms of whether the product can deliver the expected appearance, fit, and identity-expressive outcome. These perceptions further reduce consumers’ willingness to use the product for self-expression.

**Figure 1:**
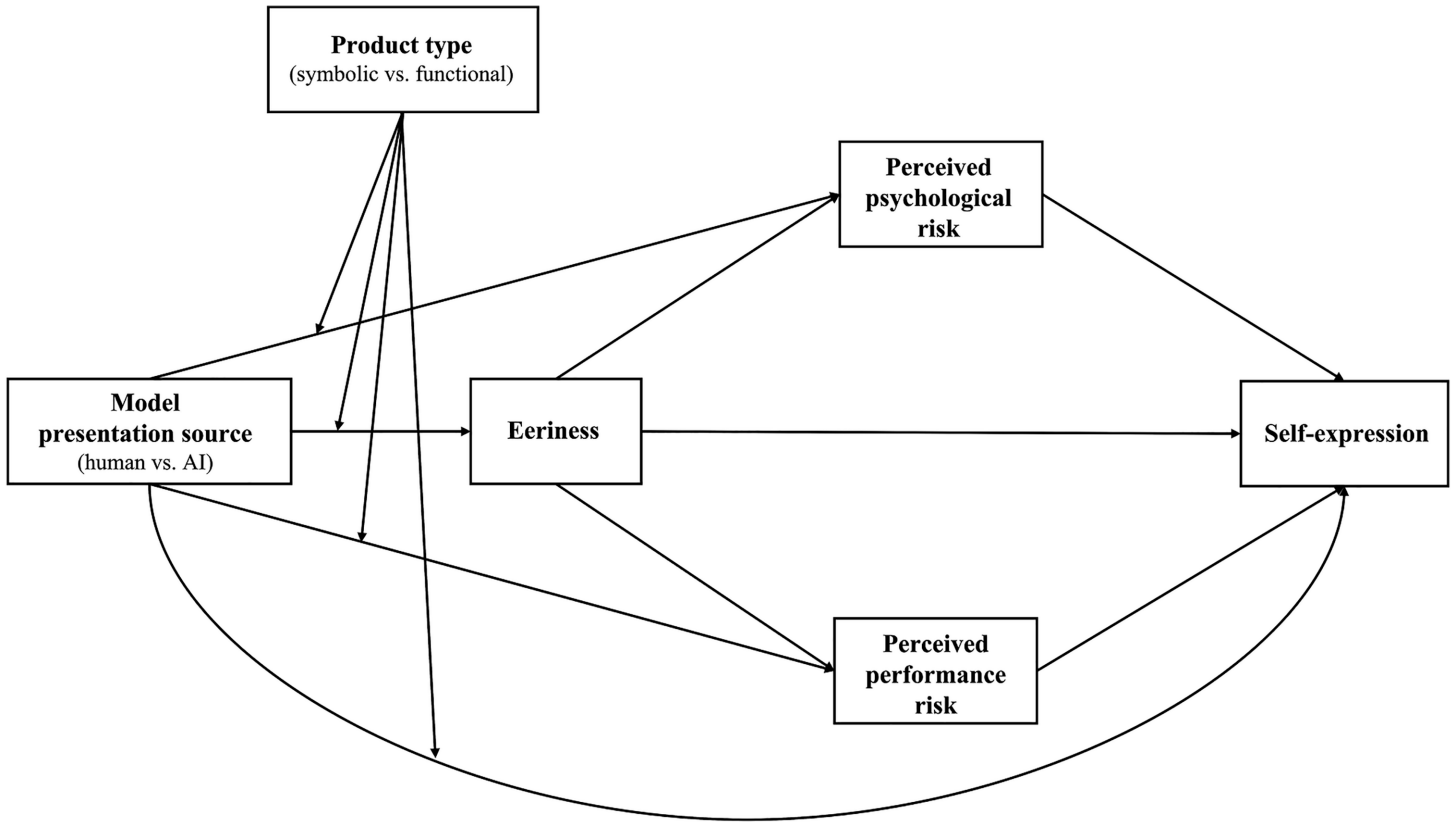
Conceptual model.

By contrast, for functional products, consumers are more likely to evaluate the product based on practical attributes (Baek and Kim, 2022). In this case, the model’s source is less central to the product’s meaning and less diagnostic for evaluating whether the product can fulfill its primary function. Thus, although the AI-generated model label still highlights the nonhuman nature of the presentation subject (Audrezet et al., 2025), its emphasis-framing effect is weaker because consumers allocate more evaluative weight to functional product attributes rather than to the model’s identity-related meaning. Consequently, the negative perceptions associated with the AI-generated model are less likely to spill over to the product, making the effects on eeriness, perceived psychological risk, perceived performance risk, and self-expression less pronounced for functional products. The conceptual model of this study is presented in Figure 1.

*H7*: Product type (functional vs. symbolic) moderates the effect of model presentation source (AI vs. human) on consumers’ psychological responses, including eeriness, perceived psychological risk, and perceived performance risk, as well as on self-expression. Specifically, when symbolic products are displayed with a model labeled as AI-generated rather than human, consumers will experience higher levels of eeriness, perceived psychological risk, and perceived performance risk, leading to lower levels of self-expression. In contrast, such effects will not emerge for functional products.

## 3 Methods

A scenario-based online experiment was conducted to test our hypotheses. An *a priori* power analysis using G*Power 3.1 indicated that a minimum of 128 participants was required (*f* = 0.25, *α* = 0.05, power = 0.80, numerator df = 1, groups = 4). To improve the stability of the statistical tests and the reliability of the results, we moderately increased the sample size during data collection and ultimately obtained 200 valid responses (Table 1).

**Table 1:** Demographic characteristics.

Items	Value	Frequency	Percentage
Gender	Male	60	30.0%
	Female	140	70.0%
Age	20s	35	17.5%
	30s	139	69.5%
	40s	16	8.0%
	50s	5	2.5%
	60s	5	2.5%
Education	High school or equivalent (e.g., GED)	15	7.5%
	Associate’s degree	4	2.0%
	Bachelor’s degree	148	74.0%
	Master’s degree	33	16.5%
Monthly income	Less than $20,000	1	0.5%
	$20,000 to 34,999	24	12.0%
	$35,000 to $49,999	24	12.0%
	$50,000 to $74,999	77	38.5%
	$75,000 to $99,999	65	32.5%
	Greater than $100,000	9	4.5%
Occupation	Full time employment (40 h or above in a week)	182	91.0%
	Part time Employment (up to 39 h per week)	8	4.0%
	Self-employed	10	5.0%
	Total	200	100.0%

To ensure greater sample uniformity and minimize procedural variations across different survey platforms, including differences in recruitment procedures, compensation rules, and task environments, all data in this study were collected through the Amazon Mechanical Turk (MTurk) platform. We selected MTurk because it offers access to a diverse pool of adult participants with experience in online tasks and digital consumption contexts (Buhrmester et al., 2011; Paolacci et al., 2010), making it suitable for the online product-presentation setting examined in this research.

All participants were residents of the United States who voluntarily took part in the experiment. Before beginning the study, they received sufficient information about the research procedure and were clearly informed that they could withdraw from the study at any stage. Given that the experimental stimuli featured female models wearing women’s apparel, female consumers were considered more contextually relevant to the purchase and evaluation setting of this product category. Therefore, a higher proportion of female participants was included in the final sample (n = 140, 70%). The sample primarily consisted of individuals in their 30s (69.5%), those holding a bachelor’s degree (74%), those with a monthly income of US$50,000 to US$75,000 (38.5%), and those employed full-time (91%).

### 3.1 Experimental design

**Figure 2:**
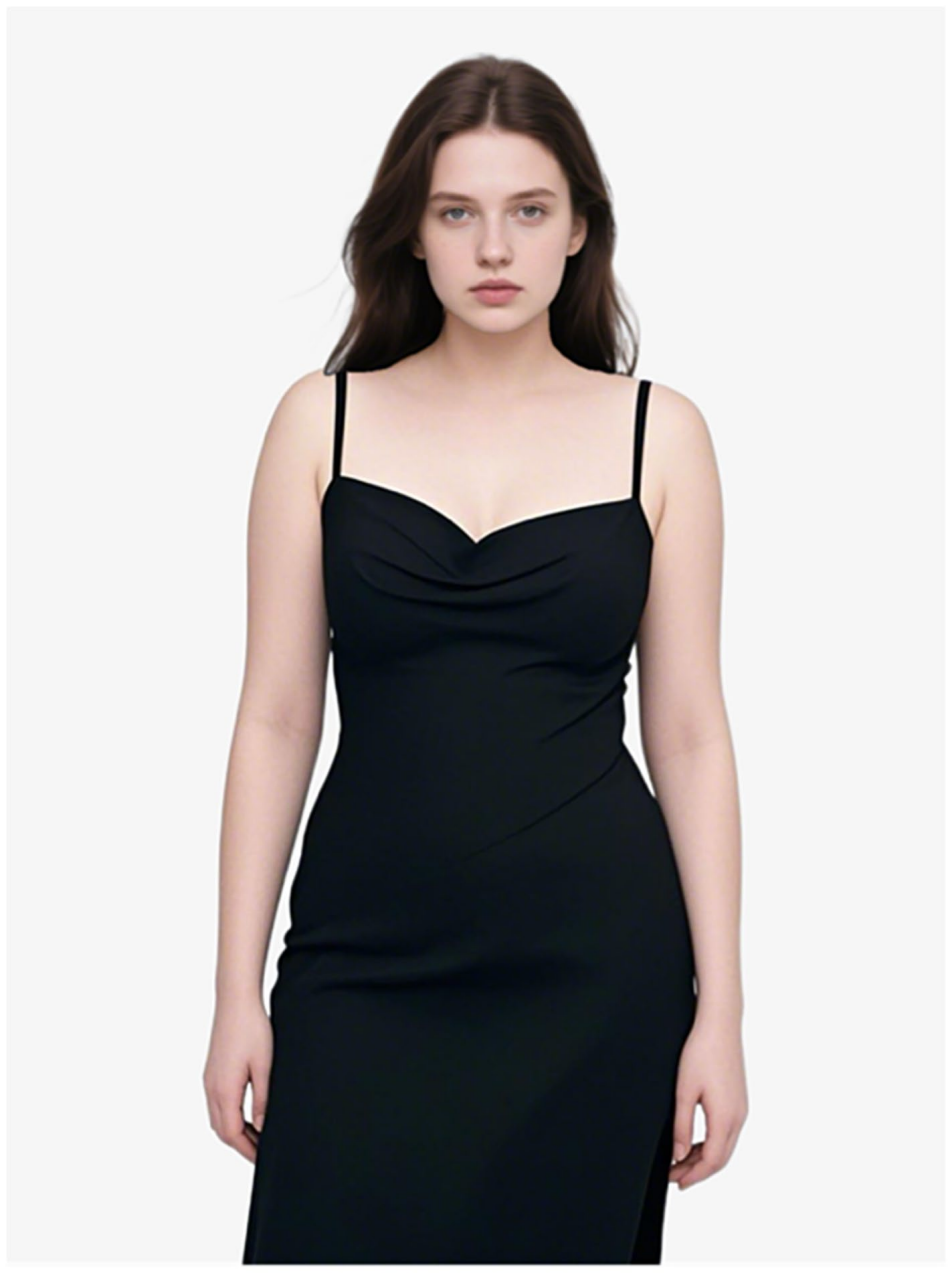
Symbolic product condition - black dress. This image was generated using Dreamina AI and is an AI-generated image used as an experimental stimulus in this study.

**Figure 3:**
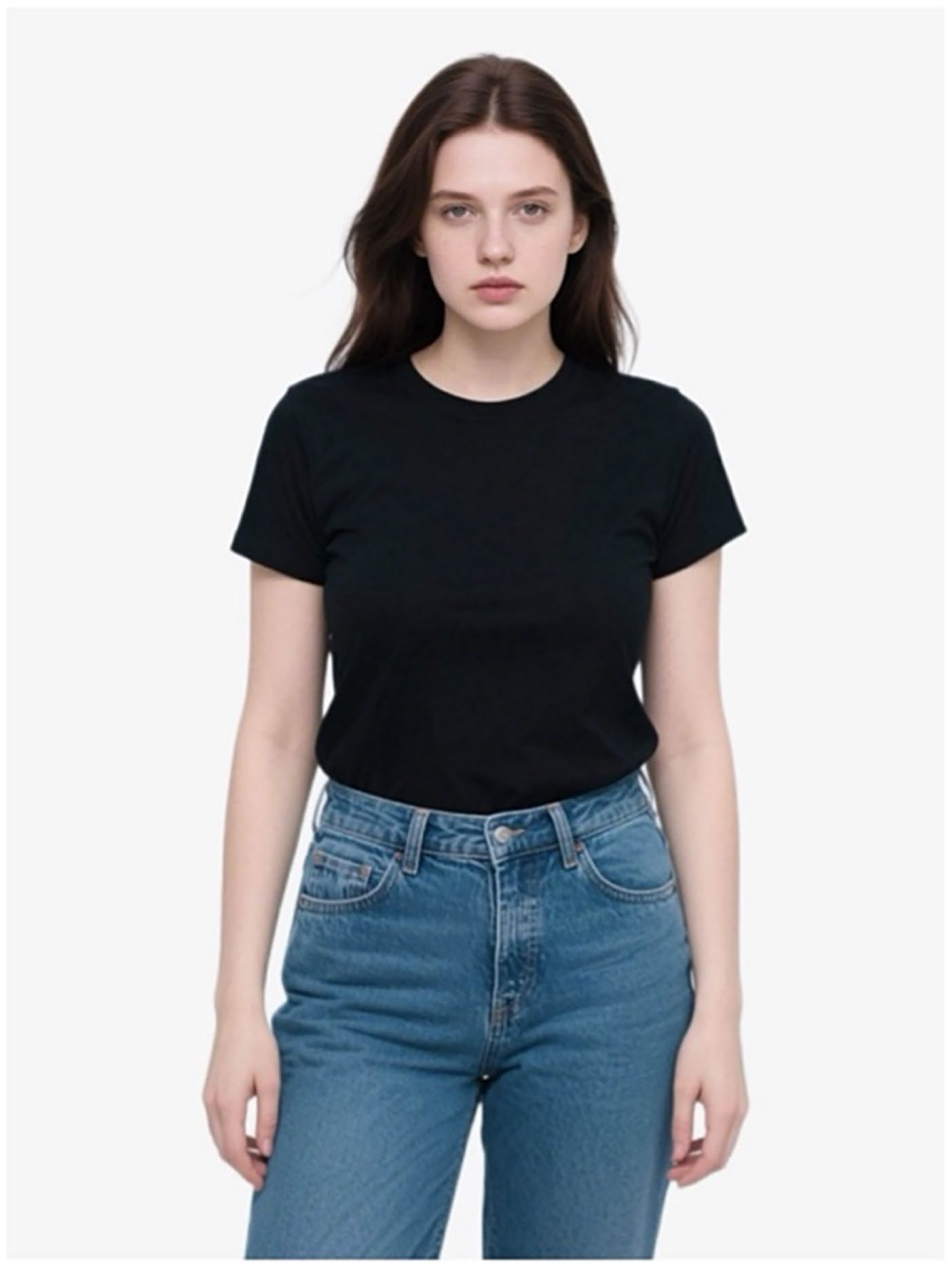
Functional product condition - black T-shirt. This image was generated using Dreamina AI and is an AI-generated image used as an experimental stimulus in this study.

This study employed a 2 (model presentation source: AI vs. human) × 2 (product type: symbolic vs. functional) between-subjects design. Participants were randomly assigned to one of the four conditions. They were first asked to read a scenario in which they imagined purchasing clothing online. In the symbolic product condition, the product was a dress (Figure 2), whereas in the functional product condition, it was a T-shirt (Figure 3). To minimize potential confounds arising from product category and color differences, both conditions used clothing items and were presented in the same black color. After reading the scenario, participants viewed a product image showing a model wearing either a dress or a T-shirt. The images were generated using the Dreamina AI tool. In the AI (human) condition, a label presented below the image indicated that the product was “displayed by an AI-generated model” (“displayed by a human model”), although the images themselves were identical across conditions.

Following exposure to the image, participants completed measures assessing the manipulation checks and the focal variables (see Table 2). Product symbolism (Cronbach’s *α* = 0.910) and functionality (Cronbach’s α = 0.868) were each measured using four items (Gilal et al., 2018). Eeriness (Cronbach’s α = 0.935) was assessed with three items (Singh et al., 2021). Perceived psychological risk (Cronbach’s α = 0.942) and perceived performance risk (Cronbach’s α = 0.912) were each measured using three items (Laroche et al., 2005). Self-expression (Cronbach’s α = 0.969) was measured using eight reverse-worded items (Breberina et al., 2022), which were subsequently reverse-coded prior to analysis.

**Table 2:** Measurements.

Variables	Measures	Factor loading	CR	AVE
Functional product	The product seems to have a good quality.	0.796	0.870	0.626
	The product seems to be reliable.	0.778		
	The product seems to be functional.	0.822		
	The product is highly durable.	0.766		
Symbolic product	The product would help me to create my unique personality.	0.863	0.911	0.718
	The product would help me to distinguish myself from others.	0.816		
	The product would help me to create distinctive image among masses.	0.842		
	The product would perfectly describe my personality.	0.868		
Eeriness	This model is creepy.	0.910	0.936	0.830
	This model is eerie.	0.917		
	This model is unnatural.	0.906		
Perceived psychological risk	The thought of purchasing this product gives me a feeling of unwanted anxiety.	0.923	0.942	0.845
	The thought of purchasing this product makes me feel psychologically uncomfortable.	0.916		
	The thought of purchasing this product causes me to experience unnecessary tension.	0.918		
Perceived performance risk	If I were to purchase this product within the next twelve months, I would become concerned that the item will not provide the level of benefits that I would be expecting.	0.874	0.912	0.776
	As I consider the purchase of this product soon, I worry about whether it will really “perform” as well as it is supposed to.	0.898		
	The thought of purchasing this product causes me to be concerned for how really reliable that product will be.	0.871		
Self-expression	You are afraid that if you buy this product, it may negatively affect what others think of you.	0.914	0.969	0.798
	You are worried that, if you buy this product, the esteem your family or friends have for you may drop.	0.854		
	You think that, if you buy this product, others will not see you the way you want them to.	0.891		
	You are afraid that, if you buy this product, others may look down on you.	0.884		
	Buying this product makes you feel unhappy or frustrated.	0.914		
	Buying this product will make you feel uncomfortable with yourself.	0.902		
	This product does not fit in well with the concept you have of yourself.	0.904		
	This product makes you doubt whether you were right in buying it.	0.883		

### 3.2 Reliability and validity assessment

We conducted a confirmatory factor analysis using AMOS to evaluate the measurement properties of the constructs. The results showed that all standardized factor loadings were above 0.75. In addition, the composite reliability values exceeded the recommended threshold of 0.70, indicating satisfactory construct reliability. The average variance extracted values were greater than 0.50, providing support for convergent validity (Table 2).

To examine discriminant validity, we compared the hypothesized four-factor model with several competing models, including three-factor, two-factor, and one-factor models (Table 3). The results indicated that the four-factor model achieved better overall fit than all alternative models and met the acceptable fit criteria (χ2 /df = 2.413, CFI = 0.964, NFI = 0.940, TLI = 0.957, RMSEA = 0.084). Further chi-square difference tests showed that the one-factor, two-factor, and three-factor models exhibited significantly poorer fit than the four-factor model. These results provide preliminary support for the discriminant validity among the four constructs.

**Table 3:** Fitting indices of competition models.

Model	χ^2^	df	χ^2^/df	NFI	CFI	RMSEA	Comparison	Δχ^2^	Δdf	P
1	573.750	119	4.821	0.874	0.897	0.139	1 vs. 4	301.118	6	0.000
2	510.009	118	4.322	0.888	0.911	0.129	2 vs. 4	237.377	5	0.000
3	338.058	116	2.914	0.926	0.950	0.098	3 vs. 4	65.426	3	0.000
4	272.632	113	2.413	0.940	0.964	0.084				

In addition, because some constructs showed relatively high correlations, we conducted additional pairwise constrained correlation tests for greater rigor (Anderson and Gerbing, 1988). Specifically, the correlation between each pair of the four constructs was constrained to 1 and compared with the corresponding unconstrained model using chi-square difference tests. The results showed that, compared with the unconstrained model, all six constrained models in which the inter-construct correlation was fixed to 1 exhibited significantly worse fit: eeriness and perceived psychological risk (Δχ^2^ = 69.819, Δdf = 1, *p* < 0.001), eeriness and perceived product performance risk (Δχ^2^ = 30.834, Δdf = 1, *p* < 0.001), eeriness and self-expression (Δχ^2^ = 235.768, Δdf = 1, *p* < 0.001), perceived psychological risk and perceived product performance risk (Δχ^2^ = 84.566, Δdf = 1, *p* < 0.001), perceived psychological risk and self-expression (Δχ^2^ = 570.151, Δdf = 1, *p* < 0.001), and perceived product performance risk and self-expression (Δχ^2^ = 362.923, Δdf = 1, *p* < 0.001). Although some constructs were highly correlated, high inter-construct correlations do not necessarily indicate complete construct overlap (Rönkkö and Cho, 2022). The results showed that model fit significantly deteriorated when the correlation between any two constructs was fixed to 1. Therefore, these constructs can still be considered empirically distinguishable.

### 3.3 Manipulation check

To assess the effectiveness of the product type manipulation, independent-samples t-tests were conducted on perceived functionality and perceived symbolism. The results indicate that, on the functionality dimension, the T-shirt received significantly higher scores than the dress [M_T-shir*t* = 5.66, SD = 0.80 vs. M_Dress = 5.29, SD = 1.39; t(158.23) = 2.32, *p* < 0.05]. Conversely, on the symbolism dimension, the dress received significantly higher scores than the T-shirt [M_Dress = 5.75, SD = 0.93 vs. M_T-shir*t* = 5.11, SD = 1.50; t(164.75) = −3.61, *p* < 0.001]. These findings confirm that the manipulation of product type was successful in this study.

### 3.4 Main effect

An independent-samples t-test was conducted to examine the main effect of model presentation source (AI vs. human) on consumers’ self-expression. The results indicate that self-expression scores in the AI model condition were significantly lower than those in the human model condition [M_AI = 3.12, SD = 1.62 vs. M_Human = 4.54, SD = 1.70; t(198) = −6.03, *p* < 0.001]. This finding suggests that AI-generated models are more likely to reduce consumers’ willingness to engage in self-expression through the product compared with human models. Therefore, H1 is supported.

In addition, before testing the mediation effects, we also conducted independent-samples t-tests to preliminarily examine differences in the mediating variables between the two groups. The results showed that the AI model group scored significantly higher than the human model group on eeriness [M_AI = 4.96, SD = 1.68 vs. M_Human = 3.76, SD = 1.99; t(192.71) = 4.62, *p* < 0.001], perceived psychological risk [M_AI = 4.81, SD = 1.76 vs. M_Human = 3.50, SD = 1.87; t(198) = 5.10, *p* < 0.001], and perceived performance risk [M_AI = 5.11, SD = 1.49 vs. M_Human = 3.95, SD = 1.75; t(192.84) = 5.04, *p* < 0.001].

### 3.5 Mediating effect

**Figure 4:**
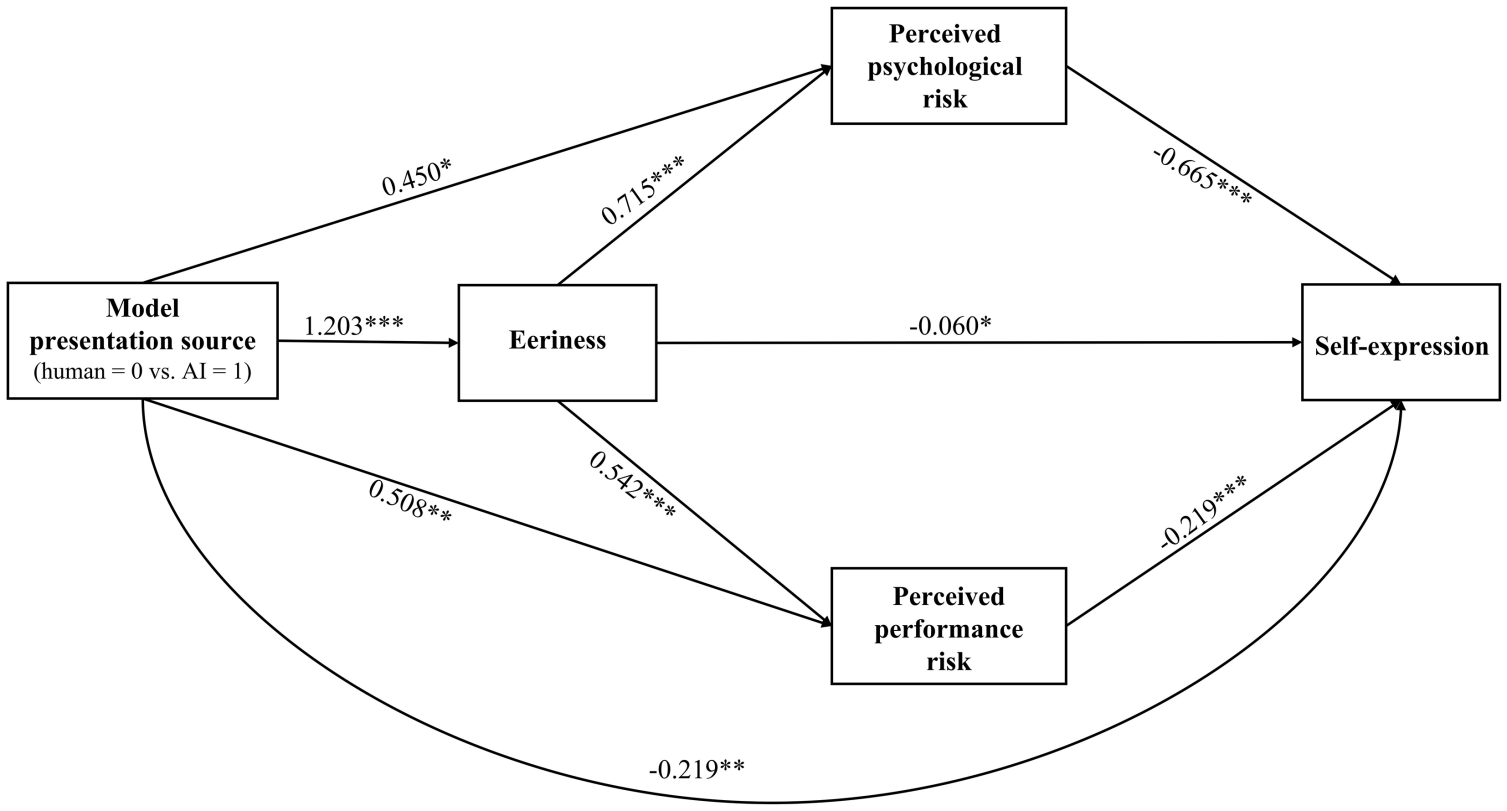
Results of the serial mediation analysis. *p < 0.05, **p < 0.01, ***p < 0.001.

We employed the PROCESS macro (Model 81) developed by Hayes (2017) PROCESS macro (Model 81) to test the mediation effects (Tables 4, 5; Figure 4). The direct effect of model presentation source (human = 0, AI = 1) on self-expression was significant [95% CI (−0.356, −0.081)]. Compared with human models, AI-generated models significantly reduced consumers’ self-expression (*β* = −0.219, *t* = −3.141, *p* < 0.01) providing further support for H1. AI-generated models significantly increased eeriness (*β* = 1.203, *t* = 4.621, *p* < 0.001), which in turn suppressed consumers’ self-expression (*β* = −0.060, *t* = −2.328, *p* < 0.05). The indirect effect of model presentation source on self-expression through eeriness was significant [95% CI (−0.166, −0.002)], supporting H2.

**Table 4:** Regression results for the mediation model.

	Model 1 (DV: EE)		Model 2 (DV: PPR)		Model 3 (DV: PPER)		Model 4 (DV: SE)	
Predictors	*β*	*t*	*β*	*t*	*β*	*t*	*β*	*t*
MPS	1.203	4.621***	0.450	2.404*	0.508	2.651**	−0.219	−3.141**
EE			0.715	14.737***	0.542	10.891***	−0.060	−2.328*
PPR							−0.665	−18.575***
PPER							−0.219	−6.264***
R^2^	0.097		0.580		0.447		0.936	
F	21.353***		135.738***		79.570***		717.943***	

MPS, model presentation source (human = 0, AI = 1); EE, eeriness; PPR; perceived psychological risk; PPER, perceived performance risk; SE, self-expression; *p < 0.05; **p < 0.01; ***p < 0.001.

**Table 5:** Bootstrap estimates of indirect effects in the serial mediation model.

Effect	Path	Effect	SE	95% Bootstrap CI
Total effect	MPS → SE	−1.416	0.235	(−1.879, −0.953)
Direct effect	MPS → SE	−0.219	0.070	(−0.356, −0.081)
Total indirect effect		−1.198	0.223	(−1.626, −0.768)
Indirect effect	MPS → EE → SE	−0.072	0.042	(−0.166, −0.002)
	MPS → PPR → SE	−0.299	0.141	(−0.594, −0.040)
	MPS → PPER → SE	−0.111	0.057	(−0.240, −0.020)
	MPS → EE → PPR → SE	−0.572	0.144	(−0.874, −0.299)
	MPS → EE → PPER → SE	−0.143	0.047	(−0.243, −0.060)

MPS = model presentation source (human = 0, AI = 1); EE = eeriness, PPR = perceived psychological risk, PPER = perceived performance risk, SE = self-expression.

In addition, the indirect effects of model presentation source on self-expression through perceived psychological risk [95% CI (−0.594, −0.040)] and perceived performance risk [95% CI (−0.240, −0.020)] were both significant. Specifically, AI-generated models increased consumers’ perceived psychological risk (*β* = 0.450, *t* = 2.404, *p* < 0.05), which subsequently reduced self-expression (*β* = −0.665, *t* = −18.575, *p* < 0.001), supporting H3. Similarly, exposure to AI-generated models significantly elevated perceived product performance risk (*β* = 0.508, *t* = 2.651, *p* < 0.01), which in turn decreased consumers’ self-expression (*β* = −0.219, *t* = −6.264, *p* < 0.001), supporting H5. Both serial mediation paths from model presentation source to self-expression through eeriness and perceived psychological risk [95% CI (−0.874, −0.299)] and through eeriness and perceived performance risk [95% CI (−0.243, −0.060)] were significant. Thus, H4 and H6 were also supported.

Overall, the negative effect of AI-generated models on consumers’ self-expression operates through multiple parallel and sequential psychological mechanisms. The direct effect remains significant, indicating that eeriness, perceived psychological risk, and perceived performance risk serve as partial mediators in the relationship between model presentation source (AI vs. human) and consumers’ self-expression.

### 3.6 Moderating effect

A two-way ANOVA was conducted to examine the moderating effect of product type. The interaction between model presentation source and product type was significant for eeriness [*F*(1, 196) = 9.05, *p* = 0.003, ηp^2^ = 0.044], perceived psychological risk [F(1, 196) = 19.55, *p* < 0.001, ηp^2^ = 0.091], perceived performance risk [F(1, 196) = 11.80, *p* < 0.001, ηp^2^ = 0.057], and self-expression [F(1, 196) = 23.10, *p* < 0.001, ηp^2^ = 0.105].

**Figure 5:**
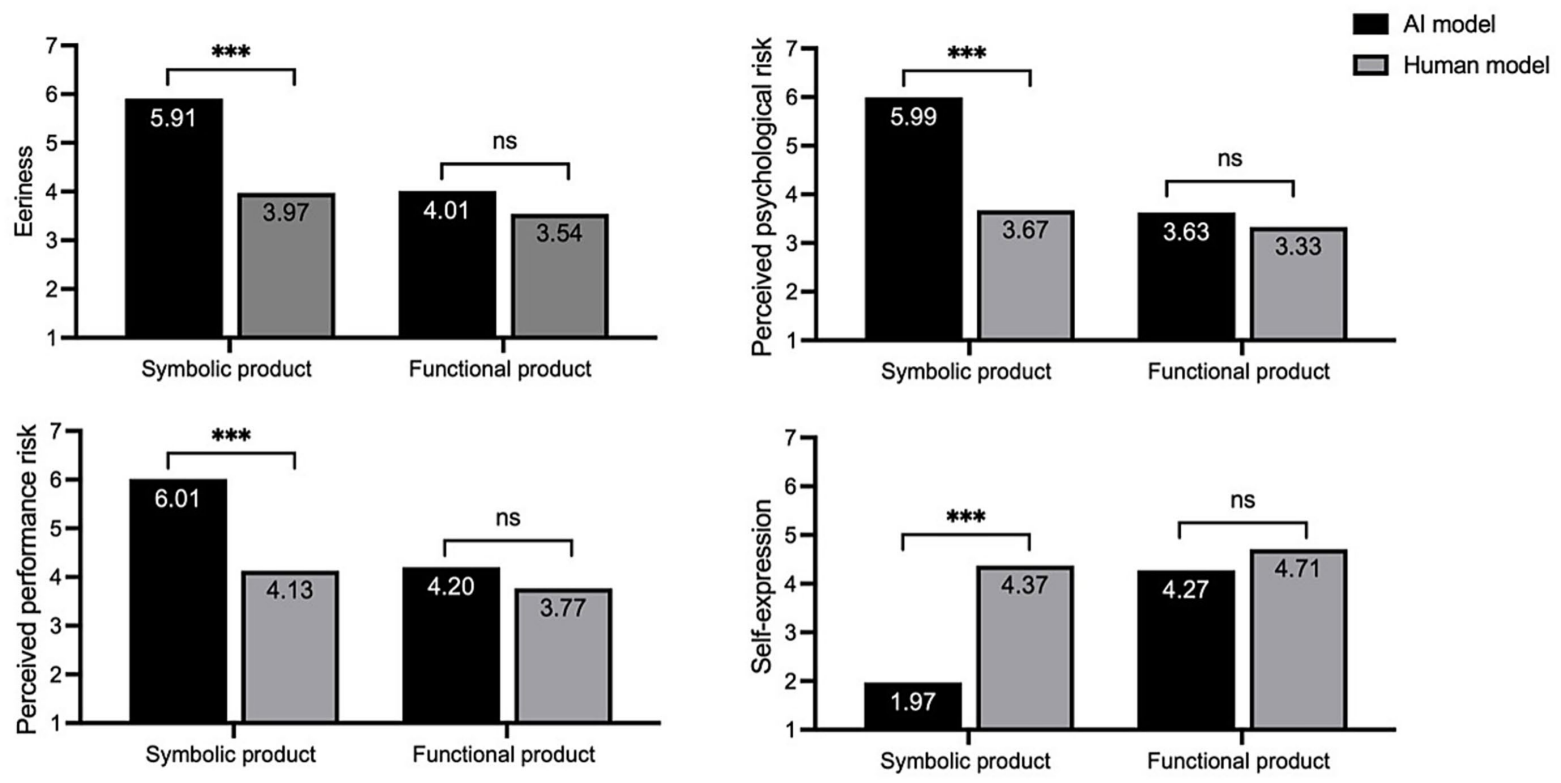
Two-way ANOVA results for the interaction of product type and model presentation source. ns; *p* > 0.05; ***p < 0.001.

To further interpret this interaction, *post hoc* comparisons between the AI model and the human model were conducted within each product type condition (Figure 5). The results indicate that, in the T-shirt (functional product) condition, there were no significant differences between the AI and human model conditions in terms of eeriness (M_AI = 4.01 vs. M_Human = 3.54, *p* = 0.169), perceived psychological risk (M_AI = 3.63 vs. M_Human = 3.33, *p* = 0.354), perceived performance risk (M_AI = 4.20 vs. M_Human = 3.77, *p* = 0.149), or self-expression (M_AI = 4.27 vs. M_Human = 4.71, *p* = 0.137).

In contrast, in the dress (symbolic product) condition, the AI model elicited significantly higher levels of eeriness (M_AI = 5.91 vs. M_Human = 3.97, *p* < 0.001), perceived psychological risk (M_AI = 5.99 vs. M_Human = 3.67, *p* < 0.001), and perceived performance risk (M_AI = 6.01 vs. M_Human = 4.13, *p* < 0.001). In addition, self-expression scores were significantly lower in the AI model condition (M_AI = 1.97 vs. M_Human = 4.37, *p* < 0.001). This suggests that AI-generated models are more likely to inhibit consumers’ self-expression in symbolic product contexts. Overall, these findings indicate that the negative effects of AI-generated models emerge only in symbolic product contexts, but not in functional product contexts, thereby supporting H7.

## 4 General discussion

### 4.1 Theoretical contributions

First, departing from prior research that has primarily examined consumers’ responses to AI-generated content itself (Jayasingh et al., 2025; Yang and Tian, 2026), this study investigates how a label disclosing that the model is AI-generated shapes consumers’ evaluations of the displayed product. Specifically, existing studies have largely treated AI as the direct object of consumer evaluation, such as whether AI influencers are perceived as credible (Jayasingh et al., 2025) or whether AI-designed products reduce consumers’ willingness to purchase (Yang and Tian, 2026). In contrast, this study shows that even when the product itself is neither designed nor manufactured by AI, merely disclosing that the model displaying the product is AI-generated can elicit eeriness. By introducing a framing perspective (Entman, 1993; Goffman, 1974; Tversky and Kahneman, 1981), this study further argues that an AI model label functions as an emphasis-framing cue (Chong and Druckman, 2007) that allows AI-related associations, such as artificiality, nonhumanness, and algorithmic generation, to spill over from the model to the displayed product. As a result, consumers may no longer evaluate the product only in terms of its appearance, quality, or price, but may also place greater weight on whether the product presentation provides a real human bodily reference and a sense of humanness. The findings suggest that when consumers are explicitly informed that a product is displayed by an AI-generated model, they may find it more difficult to project themselves into a realistic wearing context and become less willing to use the product as a means of expressing their self-image. In this way, the present research reveals a product-spillover mechanism of AI labeling, whereby negative perceptions triggered by an AI-generated presentation subject shape consumers’ subsequent product-related judgments.

Second, this study extends the concept of algorithm aversion from traditional decision-making contexts to the domain of consumers’ perceptual and emotional responses, thereby enriching its theoretical scope. Prior research has primarily focused on individuals’ acceptance or rejection of algorithmic recommendations or predictions, emphasizing that human judgment is often preferred over algorithmic output (Dietvorst et al., 2015; Filiz et al., 2023). However, such research is largely confined to adoption decisions and overlooks the emotional experiences and perceptual processing that arise when consumers are exposed to AI-generated content. This study introduces eeriness as a key first-stage mediator and finds that individuals’ aversion to algorithms not only influences their decision-making behavior but also elicits stronger negative emotional responses, namely eeriness. Moreover, by proposing a serial mediation pathway from eeriness to risk perceptions and ultimately to self-expression, this study connects affect-based explanations with perceived risk theory. The results indicate that the AI model label reduces consumers’ perception that a product can represent themselves and communicate their self-image to others by increasing eeriness and perceived psychological and performance risks, thereby lowering their tendency toward self-expression.

Finally, this study introduces product type as a key moderator, thereby clarifying the boundary conditions of the AI labeling effect. In AI marketing research, whether product type shapes consumer decision-making remains underexplored. The present findings show that the negative effects triggered by AI model labeling are particularly pronounced in symbolic product contexts, where the adverse impacts on eeriness, risk perceptions, and self-expression are significantly amplified. In contrast, these negative effects are largely absent in functional product contexts. These results suggest that consumers are not inherently resistant to the application of AI-generated technologies in commercial settings; rather, their responses depend on the type of product to which the technology is applied. For functional products, consumers tend to focus primarily on objective attributes such as specifications and quality, rendering the role of the model in product displays less critical. Under such conditions, whether the model is AI-generated, or human becomes relatively inconsequential. By contrast, for symbolic products, consumers place greater emphasis on presentation, aesthetic fit, and alignment with their self-image, making the role of the model particularly salient. In these contexts, the sense of inauthenticity and eeriness induced by AI labeling can substantially reduce consumer acceptance.

### 4.2 Practical implications

The findings also have implications for legal compliance and AI disclosure practices in digital marketing. As AI-generated images and synthetic media become increasingly common in online product presentation, regulatory frameworks in several jurisdictions have begun to emphasize transparency, disclosure, and labeling of AI-generated content (Lund et al., 2025). Although disclosure can increase transparency and reduce potential deception (Qadri and Moustafa, 2026), it may also heighten eeriness, perceived risk, and consumers’ reluctance to use the displayed product for self-expression. Therefore, firms should not treat AI-generated model labels as purely formal compliance statements. Instead, they should carefully design the wording, placement, and contextual explanation of AI labels so that transparency requirements can be met while minimizing unnecessary negative spillover to product evaluations and identity-related consumer responses. Furthermore, firms may consider consistently using a limited set of AI-generated models, assigning them names and backstories to enhance their perceived social presence. Providing additional information, such as the model’s body measurements alongside product images, may also help consumers better simulate product usage. In addition, positioning AI-generated models as brand-affiliated representatives or quasi-employees may reduce consumers’ psychological defensiveness and resistance, thereby mitigating negative reactions.

The findings further indicate that eeriness translates into both perceived psychological risk and perceived product performance risk. Therefore, firms should not focus solely on visual presentation but also reduce consumers’ perceived uncertainty through informational support. For example, providing detailed descriptions of product materials, close-up visual displays, user reviews, or real usage scenarios can help prevent consumers from transferring feelings of inauthenticity to judgments of product quality. In addition, strengthening brand trust signals, such as endorsements, quality guarantees, or after-sales commitments, can help alleviate risk perceptions driven by emotional responses.

Finally, this study highlights the critical role of product type in AI marketing contexts. For symbolic products, consumers place greater emphasis on whether the product enables self-expression and are therefore more susceptible to the negative emotional and risk-related effects triggered by AI labeling. In such contexts, firms should exercise greater caution in using AI-generated models or prioritize human models to preserve the product’s identity-expressive function. In contrast, for functional products, where consumers focus primarily on performance and utilitarian value, the negative effects of AI-generated models are relatively weaker. Consequently, firms may adopt AI-generated content more proactively in functional product contexts to achieve cost efficiency and operational effectiveness without substantial concern about adverse consumer reactions.

### 4.3 Limitations and future research

This study adopted a scenario-based experimental design, and the product-type manipulation used a dress and a T-shirt to represent symbolic and functional products, respectively. Although this design enabled us to examine product differences within the apparel category, the two stimuli may also differ in hedonic value, formality, and femininity. Therefore, future research should use more tightly controlled stimuli that account for these factors and examine whether the findings hold for male-oriented products in more realistic consumption settings.

In addition, this study focuses on eeriness as a negative emotional response; however, AI-generated content may also evoke positive emotions, such as novelty, curiosity, or technological interest. Future research could adopt a multidimensional emotional framework to investigate the roles of different emotional pathways in AI marketing. This study considers self-expression as the focal outcome variable, yet its antecedents and consequences warrant further exploration.

Finally, while this study considers product type (symbolic vs. functional) as a key boundary condition, other important contextual factors remain to be explored. For instance, cultural differences such as uncertainty avoidance, as well as brand characteristics such as brand trust and brand personality, may also influence consumers’ responses to AI-generated content. Future research could incorporate additional moderators at both the individual and contextual levels to more fully delineate the boundary conditions of AI marketing effects.

## Data Availability

The raw data supporting the conclusions of this article will be made available by the authors, without undue reservation.

## References

[ref1] Abikari M. (2024). Emotions, perceived risk and intentions to adopt emerging e-banking technology amongst educated young consumers. Int. J. Bank Mark. 42, 1036–1058. doi: 10.1108/ijbm-01-2023-0004

[ref2] Allen M. W. Gupta R. Monnier A. (2008). The interactive effect of cultural symbols and human values on taste evaluation. J. Consum. Res. 35, 294–308. doi: 10.1086/590319

[ref3] Altay S. Gilardi F. (2024). People are skeptical of headlines labeled as AI-generated, even if true or human-made, because they assume full AI automation. PNAS Nexus 3:403. doi: 10.1093/pnasnexus/pgae403 PMC11443540 39359399

[ref4] Anderson J. C. Gerbing D. W. (1988). Structural equation modeling in practice: a review and recommended two-step approach. Psychol. Bull. 103, 411–423. doi: 10.1037/0033-2909.103.3.411

[ref5] Ang S. H. Lim E. A. C. (2006). The influence of metaphors and product type on brand personality perceptions and attitudes. J. Advert. 35, 39–53. doi: 10.1080/00913367.2006.10639226

[ref6] Atwal G. Bryson D. Kaiser M. (2022). The chopsticks debacle: how brand hate flattened Dolce & Gabbana in China. J. Bus. Strateg. 43, 37–43. doi: 10.1108/jbs-07-2020-0160

[ref7] Audrezet A. Koles B. Moulard J. G. Ameen N. McKenna B. (2025). Virtual influencers: definition and future research directions. J. Bus. Res. 200:115647. doi: 10.1016/j.jbusres.2025.115647

[ref8] Baek H. Kim K. (2022). An exploratory study of consumers’ perceptions of product types and factors affecting purchase intentions in the subscription economy: 99 subscription business cases. Behavioral Sciences 12:179. doi: 10.3390/bs12060179, 35735389 PMC9220096

[ref9] Baek T. H. Kim J. Kim J. H. (2026). Effect of disclosing AI-generated content on prosocial advertising evaluation. Int. J. Advert. 45, 171–192. doi: 10.1080/02650487.2024.2401319

[ref10] Belk R. W. (1988). Possessions and the extended self. J. Consum. Res. 15, 139–168. doi: 10.1086/209154

[ref11] Bhat S. Reddy S. K. (1998). Symbolic and functional positioning of brands. J. Consum. Mark. 15, 32–43. doi: 10.1108/07363769810202664

[ref12] Bhukya R. Singh S. (2015). The effect of perceived risk dimensions on purchase intention: an empirical evidence from Indian private labels market. Am. J. Bus. 30, 218–230. doi: 10.1108/ajb-10-2014-0055

[ref13] Breberina J. Shukla P. Rosendo-Rios V. (2022). When endorsers behave badly: consumer self-expression and negative meaning transfer. Int. J. Advert. 41, 771–795. doi: 10.1080/02650487.2021.2016267

[ref14] Buhrmester M. Kwang T. Gosling S. D. (2011). Amazon's mechanical Turk: a new source of inexpensive, yet high-quality data? Perspect. Psychol. Sci. 6, 3–5. doi: 10.1177/1745691610393980, 26162106

[ref15] Candi M. Jae H. Makarem S. Mohan M. (2017). Consumer responses to functional, aesthetic and symbolic product design in online reviews. J. Bus. Res. 81, 31–39. doi: 10.1016/j.jbusres.2017.08.006

[ref16] Chandrruangphen E. Assarut N. Sinthupinyo S. (2022). The effects of live streaming attributes on consumer trust and shopping intentions for fashion clothing. Cogent Bus. Manag. 9:2034238. doi: 10.1080/23311975.2022.2034238

[ref17] Chaudhuri A. (1998). Product class effects on perceived risk: the role of emotion. Int. J. Res. Mark. 15, 157–168. doi: 10.1016/s0167-8116(97)00039-6

[ref18] Che S. Jin X. Sheng G. Lin Z. (2025). Seeking effective fit: the impact of brand-influencer fit types on consumer brand attitude. J. Retail. Consum. Serv. 84:104188. doi: 10.1016/j.jretconser.2024.104188

[ref19] Chen L. Jing K. Mei Y. (2026). The effects of brand logo and word of mouth on consumers' purchase intentions of masstige brands. J. Consum. Behav. 25, 286–302. doi: 10.1002/cb.70067

[ref20] Chen J. Liao J. (2023). The impact of oppositional loyalty on brand identification in online brand communities: the moderating role of self-expression. Curr. Psychol. 42, 26651–26662. doi: 10.1007/s12144-022-03707-6

[ref21] Chernev A. Hamilton R. Gal D. (2011). Competing for consumer identity: limits to self-expression and the perils of lifestyle branding. J. Mark. 75, 66–82. doi: 10.1509/jmkg.75.3.66

[ref22] Chong D. Druckman J. N. (2007). Framing theory. Annu. Rev. Polit. Sci. 10, 103–126. doi: 10.1146/annurev.polisci.10.072805.103054

[ref23] Çili S. Khadaroo A. (2026). “Understanding the dressed self and its motives through self and personality theories,” in Applied Psychology in Fashion: A Research-Informed Approach, (Cham: Springer Nature Switzerland), 27–61. doi: 10.1007/978-3-032-12271-1_2

[ref24] Clore G. L. Gasper K. Garvin E. (2012). “Affect as information,” in Handbook of affect and social Cognition, (New York: Psychology Press), 122–145. doi: 10.4324/9781410606181-8

[ref25] Dhar R. Wertenbroch K. (2000). Consumer choice between hedonic and utilitarian goods. J. Mark. Res. 37, 60–71. doi: 10.1509/jmkr.37.1.60.18718

[ref26] Diekhof E. K. Kastner S. Deinert D. Foerster M. Steinicke F. (2025). The uncanny valley effect and immune activation in virtual reality. Sci. Rep. 15:30473. doi: 10.1038/s41598-025-15579-4, 40830562 PMC12365102

[ref27] Dietvorst B. J. Simmons J. P. Massey C. (2015). Algorithm aversion: people erroneously avoid algorithms after seeing them err. J. Exp. Psychol. Gen. 144, 114–126. doi: 10.2139/ssrn.2466040, 25401381

[ref28] Dunham R. L. van Kleef G. A. Stamkou E. (2025). The threat of synthetic harmony: the effects of AI vs. human origin beliefs on listeners' cognitive, emotional, and physiological responses to music. Comp. Human Behav. 6:100205. doi: 10.1016/j.chbah.2025.100205

[ref29] Entman R. M. (1993). Framing: toward clarification of a fractured paradigm. J. Commun. 43, 51–58. doi: 10.1111/j.1460-2466.1993.tb01304.x

[ref30] Featherman M. S. Pavlou P. A. (2003). Predicting e-services adoption: a perceived risk facets perspective. Int. J. Hum. Comput. Stud. 59, 451–474. doi: 10.1016/s1071-5819(03)00111-3

[ref31] Filiz I. Judek J. R. Lorenz M. Spiwoks M. (2023). The extent of algorithm aversion in decision-making situations with varying gravity. PLoS One 18:e0278751. doi: 10.1371/journal.pone.0278751, 36809526 PMC9942970

[ref32] Gilal N. G. Zhang J. Gilal F. G. (2018). The four-factor model of product design: scale development and validation. J. Prod. Brand. Manag. 27, 684–700. doi: 10.1108/jpbm-11-2017-1659

[ref33] Goffman E. (1974). Frame Analysis: An Essay on the Organization of Experience. Cambridge, MA: Harvard university press.

[ref34] Gutuleac R. Baima G. Rizzo C. Bresciani S. (2024). Will virtual influencers overcome the uncanny valley? The moderating role of social cues. Psychol. Mark. 41, 1419–1431. doi: 10.1002/mar.21989

[ref35] Ha H.-Y. (2002). The effects of consumer risk perception on pre-purchase information in online auctions: brand, word-of-mouth, and customized information. J. Comput. Mediat. Commun. 8, 01–14. doi: 10.1111/j.1083-6101.2002.tb00160.x

[ref36] Hayes A. F. (2017). Introduction to Mediation, Moderation, and Conditional process Analysis: A Regression-based Approach. New York, NY: The Guilford Press.

[ref37] Helmy F. K. Soares A. M. Elmashhara M. G. Negm E. M. (2026). Understanding the use of fashion for self-expression: the role of civic engagement. J. Creat. Commun. 21, 220–238. doi: 10.1177/09732586261430188

[ref38] Ho C.-C. MacDorman K. F. (2010). Revisiting the uncanny valley theory: developing and validating an alternative to the godspeed indices. Comput. Hum. Behav. 26, 1508–1518. doi: 10.1016/j.chb.2010.05.015

[ref39] Ho C.-C. MacDorman K. F. (2017). Measuring the uncanny valley effect: refinements to indices for perceived humanness, attractiveness, and eeriness. Int. J. Soc. Robot. 9, 129–139. doi: 10.1007/s12369-016-0380-9

[ref40] Högemann M. Betke J. Thomas O. (2025). What you see is not what you get anymore: a mixed-methods approach on human perception of AI-generated images. Front. Artificial Intelligence 8:1707336. doi: 10.3389/frai.2025.1707336, 41346853 PMC12672458

[ref41] Hong I. B. (2015). Understanding the consumer's online merchant selection process: the roles of product involvement, perceived risk, and trust expectation. Int. J. Inf. Manag. 35, 322–336. doi: 10.1016/j.ijinfomgt.2015.01.003

[ref42] Jayasingh S. Sivakumar A. Vanathaiyan A. A. (2025). Artificial intelligence influencers’ credibility effect on consumer engagement and purchase intention. J. Theor. Appl. Electron. Commer. Res. 20:17. doi: 10.3390/jtaer20010017

[ref43] Kamalul Ariffin S. Mohan T. Goh Y.-N. (2018). Influence of consumers’ perceived risk on consumers’ online purchase intention. J. Res. Interact. Mark. 12, 309–327. doi: 10.1108/jrim-11-2017-0100

[ref44] Kätsyri J. Förger K. Mäkäräinen M. Takala T. (2015). A review of empirical evidence on different uncanny valley hypotheses: support for perceptual mismatch as one road to the valley of eeriness. Front. Psychol. 6:390. doi: 10.3389/fpsyg.2015.00390, 25914661 PMC4392592

[ref45] Kaur H. Anand S. (2021). Actual versus ideal self: an examination of the impact of fashion self congruence on consumer’s fashion consciousness and status consumption tendencies. J. Glob. Fash. Mark. 12, 146–160. doi: 10.1080/20932685.2020.1856705

[ref46] Kim C. K. Han D. Park S. B. (2001). The effect of brand personality and brand identification on brand loyalty: applying the theory of social identification. Jpn. Psychol. Res. 43, 195–206. doi: 10.1111/1468-5884.00177

[ref47] Kim T. H. Im H. (2026). Mental and physical humanlikeness in artificial intelligence influencers: effects on humanness, eeriness, and consumer responses. J. Consum. Behav. 25, 102–117. doi: 10.1002/cb.70060

[ref48] Kiper E. Liang Y. (2026). Do AI-generated fashion ads work as effectively as real fashion ads? An eye-tracking comparison of consumers’ visual attention. J. Glob. Fash. Mark. 17, 1–17. doi: 10.1080/20932685.2025.2548548

[ref49] Koc F. Ozkan B. Komodromos M. Efendioglu I. H. Baran T. (2025). The effects of trust and religiosity on halal products purchase intention: indirect effect of attitude. EuroMed J. Bus. 20, 141–165. doi: 10.1108/emjb-01-2024-0004

[ref50] Laroche M. Yang Z. McDougall G. H. Bergeron J. (2005). Internet versus bricks-and-mortar retailers: an investigation into intangibility and its consequences. J. Retail. 81, 251–267. doi: 10.1016/j.jretai.2004.11.002

[ref51] Li X. Christian R. G. Jiang C. (2026). That’s embarrassing: investigating identity avoidance for (in) conspicuous luxury consumers. J. Consum. Mark., 1–18. doi: 10.1108/jcm-03-2025-7765

[ref52] Li Y.-W. Yang S.-M. Liang T.-P. (2015). Website interactivity and promotional framing on consumer attitudes toward online advertising: functional versus symbolic brands. Pac. Asia J. Assoc. Inf. Syst. 7, 41–58. doi: 10.17705/1pais.07203

[ref53] Liang X. Hu X. Meng H. Jiang J. Wang G. (2022). How does model type influence consumer and online fashion retailing? Int. J. Retail Distrib. Manag. 50, 728–743. doi: 10.1108/ijrdm-05-2021-0224

[ref54] Lim S. Schmälzle R. (2024). The effect of source disclosure on evaluation of AI-generated messages. Computers Human Behavior 2:100058. doi: 10.1016/j.chbah.2024.100058, 38826717

[ref55] Lund B. Orhan Z. Mannuru N. R. Bevara R. V. K. Porter B. Vinaih M. K. . (2025). Standards, frameworks, and legislation for artificial intelligence (AI) transparency. AI Ethics 5, 3639–3655. doi: 10.1007/s43681-025-00661-4

[ref56] Mariadassou S. Klesse A.-K. Boegershausen J. (2024). Averse to what: consumer aversion to algorithmic labels, but not their outputs? Curr. Opin. Psychol. 58:101839. doi: 10.1016/j.copsyc.2024.101839, 38996629

[ref57] Miller E. J. Foo Y. Z. Mewton P. Dawel A. (2023). How do people respond to computer-generated versus human faces? A systematic review and meta-analyses. Comput. Hum. Behav. Rep. 10:100283. doi: 10.1016/j.chbr.2023.100283

[ref58] Morgan C. Townsend C. (2022). Why the drive: the utilitarian and hedonic benefits of self-expression through consumption. Curr. Opin. Psychol. 46:101320. doi: 10.1016/j.copsyc.2022.101320, 35421832

[ref59] Mori M. (1970). Bukimi no tani [the uncanny valley]. Energy 7, 33–35.

[ref60] Mori M. MacDorman K. F. Kageki N. (2012). The uncanny valley [from the field]. IEEE Robot. Autom. Mag. 19, 98–100. doi: 10.1109/MRA.2012.2192811

[ref61] Naqvi N. Shiv B. Bechara A. (2006). The role of emotion in decision making: a cognitive neuroscience perspective. Curr. Dir. Psychol. Sci. 15, 260–264. doi: 10.1111/j.1467-8721.2006.00448.x

[ref62] Nichols B. S. Schumann D. W. (2012). Consumer preferences for assimilative versus aspirational models in marketing communications: the role of product class, individual difference, and mood state. J. Mark. Theory Pract. 20, 359–376. doi: 10.2753/mtp1069-6679200401

[ref63] Ommen N. O. Heußler T. Backhaus C. Michaelis M. Ahlert D. (2010). The impact of country-of-origin and joy on product evaluation: a comparison of Chinese and German intimate apparel. J. Glob. Fashion Mark. 1, 89–99. doi: 10.1080/20932685.2010.10593061

[ref64] Pan J. (2024). How to expand the thermos cup market through network publicity and product positioning. Highlights Business Economics Management 41, 574–578. doi: 10.54097/e2njqy43

[ref65] Paolacci G. Chandler J. Ipeirotis P. G. (2010). Running experiments on Amazon mechanical Turk. J. Judgm. Decis. Mak. 5, 411–419. doi: 10.1017/s1930297500002205

[ref66] Park J. Ahn S. (2024). Traditional vs. AI-generated brand personalities: impact on brand preference and purchase intention. J. Retail. Consum. Serv. 81:104009. doi: 10.1016/j.jretconser.2024.104009

[ref67] Phamthi V. A. Nagy Á. Ngo T. M. (2024). The influence of perceived risk on purchase intention in e-commerce—systematic review and research agenda. Int. J. Consum. Stud. 48:e13067. doi: 10.1111/ijcs.13067

[ref68] Poirier S.-M. Cosby S. Sénécal S. Coursaris C. K. Fredette M. Léger P.-M. (2024). The impact of social presence cues in social media product photos on consumers’ purchase intentions. J. Bus. Res. 185:114932. doi: 10.1016/j.jbusres.2024.114932

[ref69] Qadri U. A. Moustafa A. M. A. (2026). They made it just for me! How AI transparency and influencer well-being shape consumer responses to AI-driven content. Psychol. Mark. 43, 591–608. doi: 10.1002/mar.70075

[ref70] Rönkkö M. Cho E. (2022). An updated guideline for assessing discriminant validity. Organ. Res. Methods 25, 6–14. doi: 10.1177/109442812096861

[ref71] Siddique S. Rajput A. (2022). Self-expressiveness and hedonic brand affect brand love through brand jealousy. Future Business J. 8:23. doi: 10.1186/s43093-022-00136-6

[ref72] Singh S. Olson E. D. Tsai C.-H. K. (2021). Use of service robots in an event setting: understanding the role of social presence, eeriness, and identity threat. J. Hosp. Tour. Manag. 49, 528–537. doi: 10.1016/j.jhtm.2021.10.014

[ref73] Sirgy M. J. (1982). Self-concept in consumer behavior: a critical review. J. Consum. Res. 9, 287–300. doi: 10.1086/208924

[ref74] Sirgy M. J. (1986). Self-Congruity: Toward a Theory of Personality and Cybernetics. New York: Praeger Publishers/Greenwood Publishing Group.

[ref75] Sjöberg L. (2007). Emotions and risk perception. Risk Manag. 9, 223–237. doi: 10.1057/palgrave.rm.8250038

[ref76] Spears N. Singh S. N. (2004). Measuring attitude toward the brand and purchase intentions. J. Curr. Issues Res. Advert. 26, 53–66. doi: 10.1080/10641734.2004.10505164

[ref77] Taufik D. Bouwman E. P. Reinders M. J. Noppers E. H. Dagevos H. (2022). Leveraging intrinsically rewarding symbolic attributes to promote consumer adoption of plant-based food innovations. Cleaner Responsible Consumption 4:100050. doi: 10.1016/j.clrc.2022.100050

[ref78] Thøgersen J. Jørgensen A. K. Sandager S. (2012). Consumer decision making regarding a “green” everyday product. Psychol. Mark. 29, 187–197. doi: 10.1002/mar.20514

[ref79] Tversky A. Kahneman D. (1981). The framing of decisions and the psychology of choice. Science 211, 453–458. doi: 10.1126/science.7455683, 7455683

[ref80] Upadhyay N. Kamble A. (2025). The role of environmental concerns and self-expression in ethical fashion consumption: a mediated model of consumer values. J. Glob. Mark. 38, 487–509. doi: 10.1080/08911762.2025.2472776

[ref81] van der Westhuizen L.-M. Kuhn S. W. (2024). Handmade clothing consumption as a means of self-expression. J Fashion Marketing Manag. 28, 759–774. doi: 10.1108/jfmm-07-2021-0175, 35579975

[ref82] Wang L. Zhao M. Zhang J. Wang Y. (2024). Compensatory consumption of specialty agricultural products from an ELM theory perspective: joint effect of product attributes and social affordances. Asia Pac. J. Mark. Logist. 36, 2558–2576. doi: 10.1108/apjml-09-2023-0867

[ref83] White K. Argo J. J. (2009). Social identity threat and consumer preferences. J. Consum. Psychol. 19, 313–325. doi: 10.1016/j.jcps.2009.03.007

[ref84] Wu Z. Zeng L. Huang Y. (2025). Influence of the characteristics of AI-generated advertising on consumers' purchase intention. J. Arts Cultural Studies 1:01. doi: 10.2139/ssrn.5052536

[ref85] Yang S. Li B. (2025). Study on Labubu explosion phenomenon and consumption driving mechanisms. Economics Business Management 2, 52–60. doi: 10.63313/ebm.9079

[ref86] Yang B. Sun Y. Shen X.-L. (2023). Understanding AI-based customer service resistance: a perspective of defective AI features and tri-dimensional distrusting beliefs. Inf. Process. Manag. 60:103257. doi: 10.1016/j.ipm.2022.103257

[ref87] Yang Z. Tian A. D. (2026). Designer-consumer similarity matters: the effect of AI-designed products on purchase intention. J. Retail. Consum. Serv. 90:104680. doi: 10.1016/j.jretconser.2025.104680

[ref88] Zhang L. Hur C. (2025). The impact of generative AI images on consumer attitudes in advertising. Adm. Sci. 15:395. doi: 10.3390/admsci15100395

[ref89] Zhang X. Yu X. (2020). The impact of perceived risk on consumers’ cross-platform buying behavior. Front. Psychol. 11:592246. doi: 10.3389/fpsyg.2020.592246, 33250830 PMC7673429

[ref90] Zhou T. Lu H. (2025). The effect of trust on user adoption of AI-generated content. Electron. Libr. 43, 61–76. doi: 10.1108/el-08-2024-0244

